# A Rare Case of Primary Follicular Lymphoma in the Retroperitoneum

**DOI:** 10.7759/cureus.81288

**Published:** 2025-03-27

**Authors:** Tijin Mathew, Shree Hariprasad, Teresa Varghese, Lydia George, Benjamin Easow, Parth Adrejiya, Melinda Gevorgian, Greeshma Thomas, Tehmina Zafar

**Affiliations:** 1 Internal Medicine, Southeast Health Medical Center, Dothan, USA; 2 Internal Medicine, Alabama College of Osteopathic Medicine, Dothan, USA; 3 Internal Medicine, Wellstar Spalding Medical Center, Griffin, USA; 4 Radiology, Alabama College of Osteopathic Medicine, Dothan, USA; 5 Nephrology, Southeast Health Medical Center, Dothan, USA

**Keywords:** ascites, atypical, follicular lymphoma, non-hodgkin lymphoma, retroperitoneal lymphoma

## Abstract

Primary retroperitoneal non-Hodgkin lymphoma (NHL) is considered sporadic, difficult to diagnose, and has an atypical presentation. NHL typically presents with non-tender peripheral lymphadenopathy in two-thirds of patients. Very rarely, it may present with abdominal swelling and obstructive symptoms of the respiratory and GI tracts. The primary gastrointestinal NHL accounts for 5-20% of all extra-nodal lymphomas. However, there are only a few reported cases of primary retroperitoneal lymphomas. Among them, most were diffuse large B cell lymphomas. This case is of a 54-year-old male patient who presented to the emergency department with abdominal swelling for the past four months. The CT abdomen and pelvis were indicative of massive retroperitoneal and mesenteric adenopathy with concerns for lymphoma with some volume ascites. He underwent an interventional radiology (IR)-guided retroperitoneal lymph node biopsy. Given his pleural effusion on the CT abdomen and pelvis, a CT chest was ordered, which showed loculated left-sided pleural effusion and trace pleural effusion on the right side. His lymph node biopsy results revealed grade II follicular lymphoma. This case highlights the atypical presentation of follicular lymphoma and that it should be considered in the differential diagnosis when a patient presents with pleural effusion or ascites, even though there are no B symptoms. Because of its indolence, follicular lymphoma is often diagnosed when a patient presents with obstructive symptoms and other complications like peritoneal carcinomatosis. Early diagnosis and treatment can lead to good clinical outcomes.

## Introduction

Primary retroperitoneal non-Hodgkin lymphomas (NHL) are considered sporadic and atypical presentations, thus proving to be a diagnostic challenge. Follicular lymphoma is considered to be the second-most common type of NHL, accounting for approximately 20-25% of the cases in the United States and Europe [1,2]. It can present anywhere in the body; however, based on its location, the presenting complaints may vary. Rarely five to 10% of cases may involve the retroperitoneum, often presenting with delayed diagnosis. All the scantily reported retroperitoneal lymphomas were large B-cell lymphomas [2-5]. While NHL typically presents with non-tender peripheral lymphadenopathy, it can occasionally manifest with obstructive symptoms such as pleural effusions or ascites. Here, we report a rare case of follicular lymphoma presenting as primary retroperitoneal NHL, with ascites and pleural effusion as the initial symptoms.

## Case presentation

This case involves a 54-year-old male patient with a past medical history significant only for hypertension, who presented to the emergency department with abdominal swelling for the past four months. He reported that he had abdominal distension and slight discomfort in the left upper quadrant. He had some occasional dull abdominal pain involving bilateral lower quadrants with no changes in pain characteristics with food intake or change in position. He had no significant surgical history and denied any unintentional weight loss, fever, and night sweats. He also denied any recent travel or diarrhea as well as any alcohol or smoking history. 

**Figure 1:**
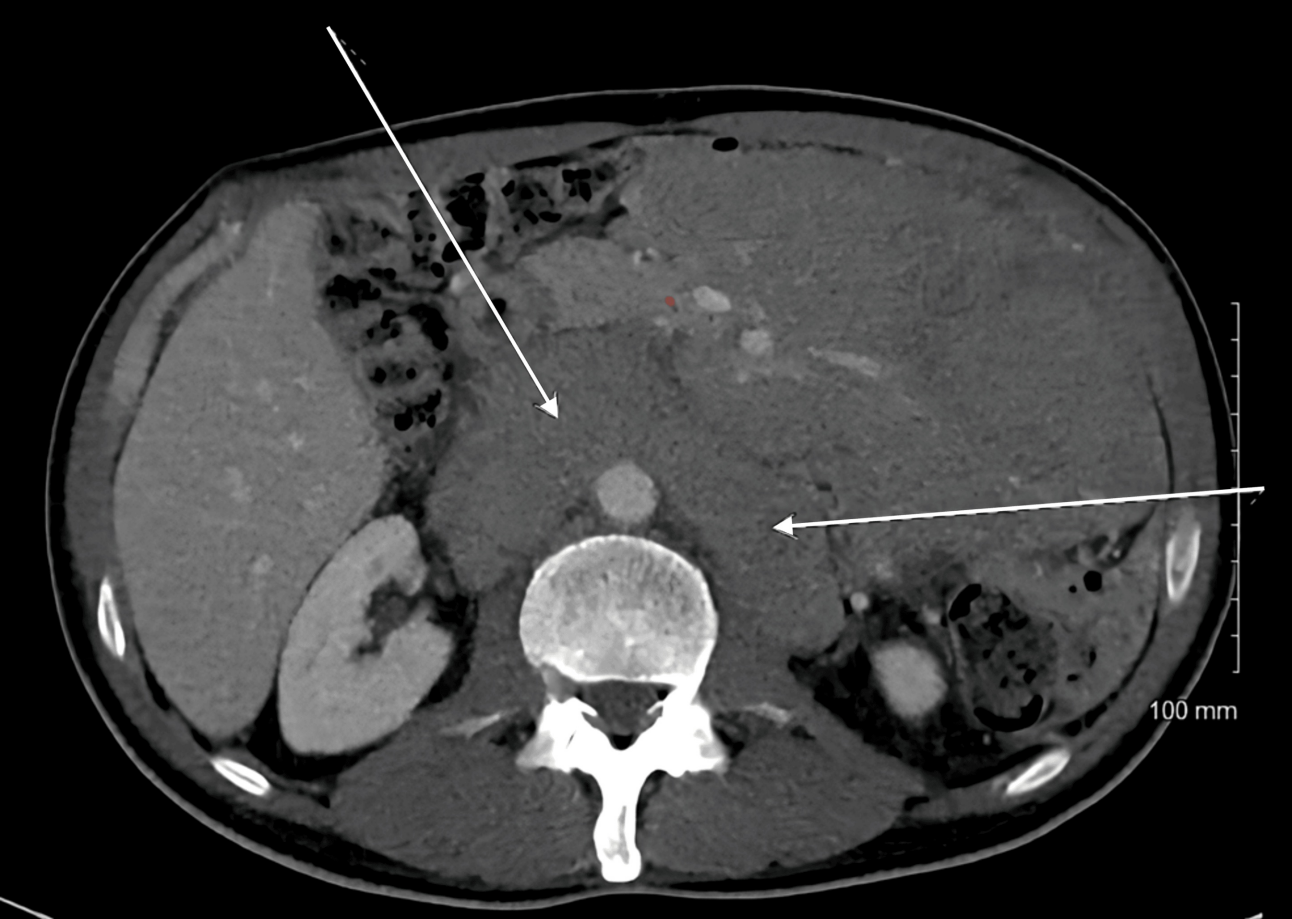
CT abdomen showing massive retroperitoneal and mesenteric adenopathy (white arrows)

The patient's vital signs were stable. The physical examination was unremarkable for any peripheral lymphadenopathy. On examination, he was found to have mild generalized abdominal distension and decreased breath sound to the left lung bases. His complete blood cell count with differential and comprehensible metabolic panel was unremarkable. The CT abdomen and pelvis were concerning for massive retroperitoneal and mesenteric adenopathy with some volume ascites (Figure 1).

**Figure 2:**
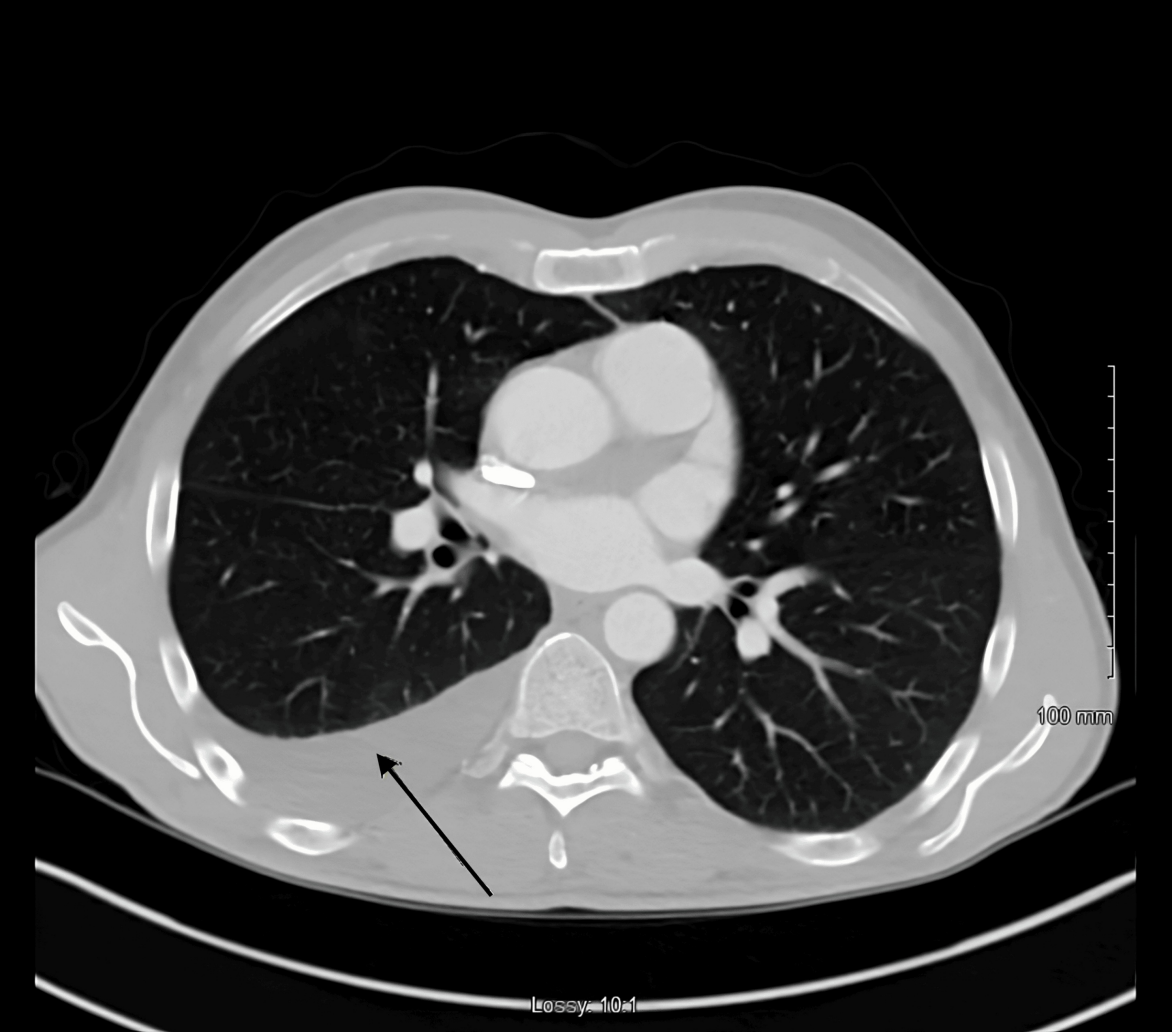
CT chest showed loculated pleural effusion on the left side and trace pleural effusion on the right (black arrow)

Given his pleural effusion on the CT abdomen and pelvis, a CT chest was ordered which showed loculated pleural effusion on the left side and trace pleural effusion on the right (Figure 2).

He underwent interventional radiology (IR)-guided thoracentesis and 300 cc of opaque white, chylous pleural fluid was drained. The pleural fluid and serum studies are listed in Table 1.

**Table 1:** Analysis of serum and pleural fluid The pleural fluid analysis was remarkable for transudate effusion. LDH: Lactate dehydrogenase

Component	Reference range	Serum	Pleural fluid	Pleural fluid/Serum
Protein (mg/dl)	6 to 8	5.3	<1.8	0.3
LDH (IU/L)	140 to 271	315	88	0.279
Triglycerides (mg/dl)	<100	N/A	2895	N/A
pH of body fluid	7.35 to 7.45	N/A	7.77	N/A

As per the Light's criteria, his etiology was considered as transudative. His differential body fluid showed 100 cells with 16% neutrophils, 53% lymphocytes, and 31% monocytes and macrophages. The pleural fluid culture was unremarkable. The patient also underwent an IR-guided retroperitoneal lymph node biopsy. The biopsy results indicate grade II follicular lymphoma with positivity for CD20, PAX5, BCL2, CD10, and BCL6, and occasional disruption of the CD21 dendritic cell meshwork. The tumor shows no cyclin D1 expression, a Ki67 proliferation index of <10%, and a low perforation rate (<10%). The patient was referred to the Hematology/Oncology department for further management. He completed six cycles of chemotherapy with bendamustine and rituximab. He tolerated the chemotherapy without any complications. On his six-month oncology follow-up, the Positron Emission Tomography (PET) scan showed complete disease resolution.

## Discussion

Primary retroperitoneal masses are often asymptomatic and present atypically, with lymphomas accounting for approximately 33% of all retroperitoneal tumors [6]. Among NHLs, diffuse large B-cell lymphoma (DLBCL) is the most common subtype, comprising 25-30% of cases, while follicular lymphoma is the second most prevalent [1,7]. Follicular lymphoma is a slow-progressing malignancy, with a median age of diagnosis of 63 years [8-10].

Chylous ascites and pleural effusion are rare manifestations of lymphoma. The proposed pathophysiological mechanism involves obstruction of the lymphatic drainage due to external compression, leading to leakage from dilated subserosal lymphatic ducts into the pleural and peritoneal cavities. Given its rare location and indolent nature, retroperitoneal follicular lymphoma is often diagnosed late despite its favorable prognosis when detected early.

Contrast-enhanced CT is the imaging modality of choice for diagnosing retroperitoneal lymphoma [8]. Additional imaging techniques include magnetic resonance imaging (MRI) and PET-CT. However, a definitive diagnosis requires tissue sampling. While excisional biopsy is the gold standard, fine needle aspiration (FNA) offers a less invasive and more time-efficient alternative [6]. In this case, FNA of the retroperitoneal mass confirmed the diagnosis of follicular lymphoma.

Follicular lymphomas can be divided into grades based on the pathology findings [9]. This classification is summarized in Table 2.

**Table 2:** Classification of follicular lymphomas based on pathology findings

Grades of the follicular lymphomas	Pathology findings
Grade 1	Follicular small cleaved cell lymphoma with 0 to 5 centroblasts per high-power field (HPF)
Grade 2	Follicular mixed–cell lymphoma with 6 to 15 centroblasts per HPF
Grade 3	Follicular large cell lymphoma with >15 centroblasts per HPF
Grade 3 A	Centrocytes present
Grade 3 B	Solid sheets of centroblasts

Treatment options vary based on the stage and the biological behavior of the disease, whether indolent or aggressive. Management strategies include radiotherapy, immunochemotherapy (rituximab plus chemotherapy), the R-CHOP (rituximab, cyclophosphamide, doxorubicin, vincristine, and prednisone) regimen, single-agent rituximab, the CVP (cyclophosphamide, vincristine, and prednisolone) regimen, and combined immunochemotherapy with radiotherapy [10]. In select cases, surgical resection may be considered for localized disease or to alleviate tumor-related symptoms. In Stage I and Grades 1, 2, and 3, radiotherapy alone is preferred [9,10]. With advanced stages and grades, the patient needs aggressive treatment with immunochemotherapy like R-CHOP [9,10].

## Conclusions

This case highlights an atypical presentation of primary retroperitoneal follicular lymphoma with chylous ascites and pleural effusion, a rare manifestation that can delay diagnosis. Given its indolent nature, early recognition of follicular lymphomas in unusual locations, even in the absence of B symptoms, is critical for timely intervention. Imaging modalities such as contrast-enhanced CT and PET-CT are integral to the initial evaluation. However, a definitive diagnosis requires histopathological confirmation via biopsy. Treatment is specific to the disease stage and progression, with immunochemotherapy remaining the mainstay of management. This case underscores the importance of including lymphomas in the differential diagnosis of unexplained pleural effusion or ascites and emphasizes the need for prompt oncologic evaluation to optimize patient outcomes.
